# Localized Retinal Nerve Fiber Layer Defects Detected by Optical Coherence Tomography: The Beijing Eye Study

**DOI:** 10.1371/journal.pone.0068998

**Published:** 2013-07-22

**Authors:** Liang Zhao, Ya Xing Wang, Wei Zhang, Jing Shang Zhang, Chang Xi Chen, Liang Xu, Jost B. Jonas

**Affiliations:** 1 Beijing Institute of Ophthalmology, Beijing Tongren Hospital, Capital University of Medical Science, Beijing, China; 2 Department of Ophthalmology, Medical Faculty Mannheim of the Ruprecht-Karls-University Heidelberg, Germany; The University of Melbourne, Australia

## Abstract

**Objective:**

To assess the prevalence of localized retinal nerve fiber layer defects (LRNFLD) and associated factors in adult Chinese.

**Methods:**

The population-based Beijing Eye Study 2011 included 3468 individuals (mean age: 64.6±9.8 years (range: 50–93 years)). The study participants underwent a detailed ophthalmological examination including spectral-domain optical coherence tomography (Spectralis^R^-OCT) assisted measurement of the RNFL. A LRNFLD was defined as a sector in which the RNFL contour line dipped into the red zone for a length of <180°.

**Results:**

Readable OCT images were available for 3242 (93.5%) subjects. LRNFLDs were detected in 640 eyes (9.9±0.4%) of 479 subjects (14.8±0.6%). In the age groups of 50–59 years, 60–69 years, 70–79 years, and 80+ years, the prevalence of LRNFLD per person increased from 9.9±0.9%, 11.6±1.0% and 20.6±1.4% to 33.0±3.2%, respectively. In multivariate analysis, prevalence of LRNFLDs was significantly associated with older age (*P* = 0.001; Odds Ratio (OR): 1.03; 95% Confidence Interval (CI): 1.01,1.05), myopic refractive error (*P*<0.001;OR:0.79;95%CI:0.74,0.85), larger beta zone of parapapillary atrophy (*P*<0.001; OR:1.34;95%CI:1.20,1.50), presence of glaucomatous optic neuropathy (*P*<0.001;OR:7.02;95%CI:3.87,12.7), presence of non-glaucomatous optic nerve damage (*P* = 0.001;OR:43.3;95%CI:8.24,227.1), and presence of diabetic retinopathy (*P* = 0.003;OR:2.79;95%CI:1.43,5.44).

**Conclusions:**

OCT-defined LRNFLDs were present in a prevalence of 14.8±0.6% in a population-based study sample of subjects aged 50+ years. Prevalence of LRNFLDs increased with higher age, myopic refractive error, and larger parapapillary beta zone. Major ocular diseases associated with LRNFLs were glaucoma, non-glaucomatous optic nerve damage and diabetic retinopathy. These data may be helpful for a semiautomatic assessment of the RNFL.

## Introduction

The assessment of the retinal nerve fiber layer (RNFL) is of high importance for the diagnosis and follow-up of any optic nerve abnormality and disease since the optic nerve fibers are the continuation of the retinal nerve fibers to the lateral geniculate ganglion [1], [2]. A loss in the RNFL layer has been divided into a diffuse loss and localized defects [3], [4]. Since a sharply delineated localized RNFL defect (RNFLD) does not occur in normal eyes, it has a high predictive value for abnormality [5]. The reasons for a localized RNFLDs are manifold and include, besides glaucomatous optic neuropathy, diabetic retinopathy and other disease leading to retinal cotton-wool spots, optic disc drusen, nonarteritic anterior ischemic optic neuropathy, pituitary gland tumors, and other causes [6]. After the clinical introduction of optical coherence tomography (OCT) by Huang and colleagues, the newly developed spectral-domain OCT technology has further improved the visualization of the RNFL and the detection of its defects [7]–[13]. Applying the spectral-domain OCT technology in a population-based study, we examined the RNFL in a cohort of more than 3000 study participants, assessed the prevalence of localized RNFLDs and searched for associations of localized RNFLDs with other ocular and systemic parameters. Knowledge about the prevalence of localized RNFLDs and the associated factors in a population is of clinical importance to assess sensitivity, specificity and diagnostic precision of the occurrence of localized RNFLDs for the detection of diseases such as glaucoma. Since the OCT examination is a non-invasive method, the results of our study could also be helpful for a semi-automatic detection of diseases associated with localized RNFLDs.

## Methods

### Ethics Statement

The Medical Ethics Committee of the Beijing Tongren Hospital approved the study protocol and all participants gave informed written consent, according to the Declaration of Helsinki.

The Beijing Eye Study 2011 is a population-based cross-sectional study in Northern China. It was carried out in 5 communities in an urban district in the North of Central Beijing and in 3 communities in a rural region south of Beijing. The study was described in detail previously [14], [15]. All people living in the 8 communities were officially registered by name, gender and age at the local mayor’s office. Using this register as the sampling frame, all subjects living in the communities and fulfilling the inclusion criterion of an age of 50+ years were eligible for the study. Home visits were performed according to the registration list, and the eligibility criteria for the study, an age of 50 or more years, was confirmed by door-to-door enrollment. Out of an eligible population of 4403 individuals fulfilling the only inclusion criterion of an age of 50+ years, 3468 (78.8%) individuals (1963 (56.6%) women) participated in the eye examination. The study was divided into a rural part (1633 (47.1%) subjects; 943 (57.7%) women) and an urban part (1835 (52.9%) subjects; 1020 (55.6%) women). The mean age was 64.6±9.8 years (median, 64 years; range, 50–93 years).

Trained research technicians asked the study participants questions on demographic variables, socioeconomic background, and known major systemic diseases. Cognitive function was assessed using the MMSE (mini mental state examination) scale [16]. Fasting blood samples were taken for measurement of blood lipids, glucose and glycosylated hemoglobin HbA1c. Blood pressure was measured. Body height and weight and the circumference of the waist and hip were recorded. The ophthalmic examination included measurement of presenting visual acuity and uncorrected visual acuity. Best corrected visual acuity was assessed by automatic refractometry (Auto Refractometer AR-610, Nidek Co., Ltd, Tokyo, Japan), if uncorrected visual acuity was lower than 1.0. Intraocular pressure was measured. A slit lamp examination carried out by an experienced ophthalmologist assessed lid abnormalities, Meibomian gland dysfunction, corneal disorders, and peripheral anterior chamber depth using van Herick’s method. Using optical low-coherence reflectometry (Lensstar 900® Optical Biometer, Haag-Streit, 3098 Koeniz, Switzerland), biometry of the right eyes was performed for measurement of the anterior corneal curvature, central corneal thickness, anterior chamber depth, lens thickness and axial length. The pupil was dilated using tropicamide once or twice, until the pupil diameter was at least 6 mm. Digital photographs of the cornea and lens and retro-illuminated photographs of the lens were taken using the Neitz CT-R camera (Neitz Instruments Co., Tokyo, Japan). Monoscopic photographs of the macula and optic disc were obtained using a fundus camera (Type CR6-45NM, Canon Inc. U.S.A.).

Glaucoma was defined according to the criteria of the International Society of Geographic and Epidemiological Ophthalmology ISGEO [17]. Mean ocular perfusion pressure was calculated as: 2/3 (diastolic blood pressure +1/3 (systolic blood pressure – diastolic blood pressure)) – intraocular pressure. Non-glaucomatous optic nerve damage was defined by increased optic nerve head pallor with a normal shape of the neuroretinal rim according to the Inferior-Superior-Nasal-Temporal-(ISNT)-rule, and a reduced retinal arteriole diameter [18]. Diabetic retinopathy was characterized according to the criteria of the Early Treatment of Diabetic Retinopathy Study (ETDRS) [19]. For the assessment of age-related macular degeneration, the International ARM (Age-related Maculopathy);Epidemiological Study Group Grading system was used [20]. The area of the optic disc, neuroretinal rim and alpha one and beta zone of parapapillary atrophy were measured on optic disc photographs [21] taking into account the interindividual differences in optic disc dimensions [22].

**Figure 1:**
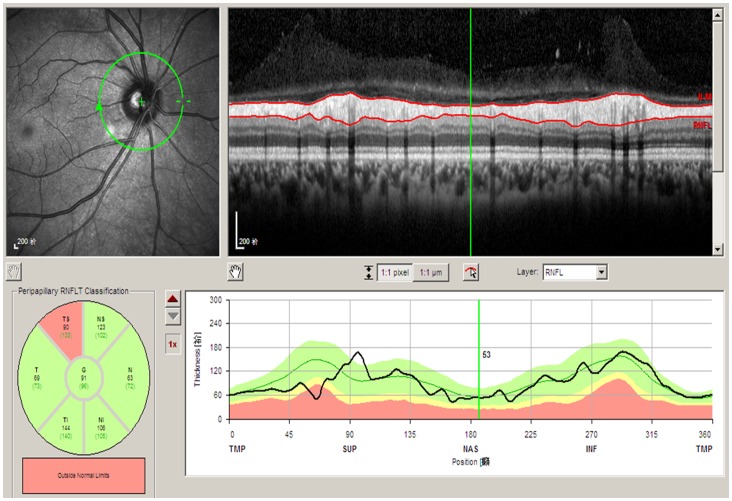
Optical Coherence Tomograph Printout of the Retinal Nerve Fiber Layer Thickness Contour Line Showing a Localized Retinal Nerve Fiber Layer Defect (Arrow).

For all study participants, the RNFL thickness was measured by the spectral domain OCT (Spectralis HRA+OCT, Heidelberg Engineering, Heidelberg, Germany). We used the high-speed resolution mode to collect the images. Circular B-scans (3.4-mm diameter, 768 pixels, 1536 A-scans/second) centered at the optic disc were automatically averaged to reduce speckle noise. The pupil was dilated by the 1% tropicamide before examination. The Spectralis OCT software, Heidelberg Explorer (HEE, version 5.3) was used for the automatic segmentation of the RNFL and for the calculation of the RNFLT. All automatically generated outlining of the RNFL was controlled and the upper border and the lower border of the RNFL were interactively re-adjusted when deemed necessary by the examiner. All RNFL assessments were performed by trained examiners (LZ, YXW) in a masked manner with knowledge of any other data than the OCT image. The Spectralis OCT software provided a classification based on the comparison with an internal normative database. The yellow zone in the printout of the RNFL thickness contour line represented the 95%–99% confidence interval of the normal RNFL thickness according to the internal normal database (Fig. 1). The red zone on the printout showed a RNFL thickness outside of the 99% confidence interval. A localized RNFLD was defined as a sector in which the RNFL contour line touched or dipped into the red zone for a length of less than 180°. The middle point of the sector was taken to describe its location. A diffuse RNFL loss was defined by an affected sector with a width of more than 180°. The present study addressed localized RNFLD only.

Only those subjects with OCT measurements of the RNFL thickness were included into the study. Statistical analysis was performed using a commercially available statistical software package (SPSS for Windows, version 20.0, IBM-SPSS, Chicago, IL). In a first step, we determined the mean values (presented as mean ± standard deviation) of the main parameters. The frequency parameters were presented as mean ± standard error. In a second step, we performed univariate analyses of the associations between the prevalence of RNFLDs and other ocular parameters and systemic parameters. In a third step, we carried out multivariate regression analyses with the RNFLDs as the dependent parameter and with all those variables as independent parameters that were significantly associated with RNFLDs in the univariate analyses. Odds ratios (OR) and 95% confidence intervals (CI) were presented. *P*-values were two-sided. A *P*-value <0.05 was considered to indicate statistical significance.

## Results

Out of the 3468 participants, 3368 participants underwent the OCT examination. Due to unreliable RNFL measurements, 126 subjects were excluded, so that eventually 3242 (93.5%) subjects were included into the study. For 3199 subjects, bilateral OCT images were available. Mean age was 64.4±9.6 years (median: 63 years; range: 50 to 93 years), mean refractive error (spherical equivalent) was −0.17±1.96 diopters (median: 0.25 diopters; range: −22.0 to +7.00 diopters), and mean axial length was 23.2±1.1 mm (median: 23.1 mm; range: 18.96 mm –30.88 mm). The group of subjects without OCT measurements as compared with the group of subjects with OCT measurements was significantly older (67.8±12.1 years versus 64.4±9.6 years; *P*<0.001), was more myopic (−1.61±4.53 diopters versus −0.17±1.96 diopters; *P* = 0.001), had a longer axial length 23.9±2.0 mm versus 23.2±1.1 mm; *P* = 0.002), had a significantly higher percentage of women (64.6% versus 56.0%; *P* = 0.01), and lived in a higher percentage in the rural region (58.0% versus 46.3%; *P* = 0.001). Reasons why OCT images were not available were opacities of the optic media such as cataract, and insufficient quality of the images for a reliable determination of the RNFL. That included cases in which the RNFL upper and lower borders could only poorly be identified, decentration of the image, or algorithm failures [23], [24].

Localized RNFLDs were detected in 640 eyes (prevalence rate: 9.9±0.4%) of 479 subjects (14.8±0.6%). The prevalence of the localized RNFLDs was significantly (*P*<0.001) higher in the superior quadrant (4.0±0.3%) and in the inferior quadrant (3.6±0.3%) than in the temporal quadrant (1.8±0.2%) and the nasal quadrant (1.5±0.2%). The mean width of a RNFLD was 47.6±58.3° (median: 24.6°).

In univariate analysis, localized RNFLDs were associated with higher age (*P*<0.001). In the age groups of 50–59 years, 60–69 years, 70–79 years, and 80+ years, the prevalence of RNFLD per person increased from 9.9±0.9%, 11.6±1.0% and 20.6±1.4% to 33.0±3.2%, respectively. In univariate analysis, prevalence of localized RNFLDs were associated with the systemic parameters of male gender (*P*<0.001), urban region of habitation (*P*<0.001), lower cognitive score (*P*<0.001), lower diastolic blood pressure (*P* = 0.002), lower prevalence of reported snoring (*P* = 0.03), higher prevalence of diabetes mellitus (*P* = 0.02) and dyslipidemia (*P* = 0.02); and with the ocular parameters of lower best corrected visual acuity (*P*<0.001), myopic refractive error (*P*<0.001), longer axial length (*P*<0.001), thicker central cornea (*P* = 0.02), deeper anterior chamber (*P*<0.001), smaller neuroretinal rim area (*P*<0.001), lower average retinal nerve fiber layer thickness (*P*<0.001), thinner subfoveal choroid (*P*<0.001), larger beta zone of parapapillary atrophy (*P*<0.001), presence of optic disc hemorrhage (*P*<0.001), prevalence of glaucoma in general (*P*<0.001), and open-angle glaucoma (*P*<0.001) and angle-closure glaucoma (*P*<0.001) in particular (Table 1).

**Table 1:** Associations (Univariate Analysis) between the Prevalence of Localized Retinal Nerve Fiber Layer Defects and Ocular and Systemic Parameters in the Beijing Eye Study 2011.

Parameters	*P*-Value	RegressionCoefficient		Odds Ratio	95% Confidence Interval	
Age (Years)	<0.001	0.05		1.05	1.04	1.06
Gender (Men/Women)	<0.001			0.67	0.57	0.79
Rural/urban Region	<0.001			1.45	1.23	1.72
Body Height(cm)	0.68					
Body Weight(kg)	0.20					
Body Mass Index (kg/m^2^)	0.06	−0.03		0.97	0.94	1.00
Cognitive scores	<0.001	−0.07		0.93	0.90	0.96
Systolic Blood Pressure (mmHg)	0.29					
Diastolic Blood Pressure (mmHg)	0.002	−0.01		0.99	0.98	0.996
Mean Arterial Blood Pressure (mmHg)	0.72					
Ocular Perfusion Pressure (mmHg)	0.72					
Blood concentration of						
HbA1C (%)	0.065	0.10		1.11	0.99	1.23
Cholesterol (mmol/L)	0.54					
Creatinine(mmol/L)	0.58					
CRP (mmol/L)	0.65					
Glucose (mmol/L)	0.89					
High-Density Lipoproteins (mmol/L)	0.40					
low-Density Lipoproteins (mmol/L)	0.67					
Triglyceride(mmol/L)	0.35					
Dyslipidemia	0.02			1.41	1.06	1.88
Diabetes Mellitus	0.02			1.83	1.15	2.93
Smoke history	1.00					
Smoking Package Years	0.75					
Snoring (No, Moderate, Severe)	0.03	−0.20		0.82	0.69	0.98
Best Corr. Visual Acuity (logMAR)	<0.001	1.18		3.25	2.59	4.07
Refractive error (diopter)	<0.001	−0.27		0.76	0.73	0.79
Axial Length (mm)	<0.001	0.44	1.55		1.41	1.71
Cornea Curvature Radius	0.52					
Central Cornea Thickness (µm)	0.02	0.004	1.004		1.001	1.008
Anterior Chamber Depth (mm)	<0.001	0.54	1.72		1.41	2.09
Lens Thickness (mm)	0.35					
Intraocular Pressure (mmHg)	0.34					
Optic Disc Area (mm^2^)	0.17					
Neuroretinal Rim Area (mm^2^)	<0.001	−1.51	0.22		0.15	0.32
Optic Disc Hemorrhage	<0.001		4.18		2.27	7.69
Parapapillary Atrophy, Alpha Zone (mm^2^)	0.19					
Parapapillary Atrophy, Beta Zone (mm^2^)	<0.001	0.46	1.58		1.40	1.78
Average Retinal Nerve Fiber Layer Thickness (µm)	<0.001	−0.11	0.90		0.88	0.91
Subfoveal Choroidal Thickness (µm)	<0.001	−0.005	0.995		0.994	0.996
Glaucoma	<0.001		10.1		7.78	13.1
Open-Angle Glaucoma	<0.001		10.5		7.72	14.3
Angle-Closure Glaucoma	<0.001		7.18		4.56	11.3
Non-Glaucomatous Optic Neuropathy	<0.001		32.3		13.7	75.8
Diabetic Retinopathy	<0.001		3.26		1.90	5.59
Age-Related Macular Degeneration	0.89					

In the ensuing multivariate analysis, prevalence of localized RBFLDs was taken as the dependent parameter. Independent parameters were all variables which were significantly associated with localized RNFLDs in the univariate analysis. The analysis revealed that localized RNFLDs remained to be significantly associated with higher age (*P* = 0.001), myopic refractive error (*P*<0.001), larger beta zone of parapapillary atrophy (*P*<0.001), presence of glaucomatous optic neuropathy (*P*<0.001), presence of non-glaucomatous optic nerve damage (*P* = 0.001), and presence of diabetic retinopathy (*P* = 0.003) (Table 2). If overall RNFL thickness was added to the multivariate analysis, prevalence of localized RNFLDs were associated with the ocular parameters of myopic refractive error (*P* = 0.03), thinner overall retinal nerve fiber layer (*P*<0.001), larger beta zone of parapapillary atrophy (*P* = 0.02), presence of glaucomatous optic neuropathy (*P*<0.001), presence of non-glaucomatous optic nerve damage (*P* = 0.001), and presence of diabetic retinopathy (*P* = 0.02) (Table 3).

**Table 2:** Associations (Multivariate Analysis) between the Prevalence of Localized Retinal Nerve Fiber Layer Defects and Ocular Parameters (Excluding Overall Retinal Nerve Fiber Layer Thickness) and Systemic Parameters in the Beijing Eye Study 2011.

Parameters	*P*-Value	Regression Coefficient	Odds Ratio	95% Confidence Interval	
Age (Years)	0.001	0.03	1.03	1.01	1.05
Refractive Error (Diopter)	<0.001	−0.23	0.79	0.74	0.85
Parapapillary Atrophy Zone Beta Area (mm^2^)	<0.001	0.29	1.34	1.20	1.50
Glaucomatous Optic Neuropathy	<0.001	1.95	7.02	3.87	12.7
Non-Glaucomatous Optic Nerve Damage	<0.001	3.77	43.3	8.24	227.1
Diabetic Retinopathy	0.003	1.03	2.79	1.43	5.44

**Table 3:** Associations (Multivariate Analysis) between the Prevalence of Localized Retinal Nerve Fiber Layer Defects and Ocular Parameters (Including Overall Retinal Nerve Fiber Layer Thickness) and Systemic Parameters in the Beijing Eye Study 2011.

Parameters	*P*-Value	Regression Coefficient	Odds Ratio	95% Confidence Interval	
Refractive Error (Diopter)	0.03	−0.09	0.91	0.84	0.99
Retinal Nerve Fiber Layer Thickness (µm)	<0.001	−0.14	0.87	0.85	0.89
Parapapillary Atrophy Zone Beta Area (mm^2^)	0.02	0.16	1.18	1.03	1.34
Glaucomatous Optic Neuropathy	<0.001	1.40	4.04	1.98	8.26
Non-Glaucomatous Optic Nerve Damage	0.001	3.06	21.4	3.33	136.7
Diabetic Retinopathy	0.02	1.01	2.75	1.16	6.53

To account for intra-individual inter-eye dependencies which might have resulted in an overestimation of the associations between LRNFLD and systemic parameters, we selected one eye randomly per subject and repeated the statistical analysis. It revealed that localized RNFLDs remained to be significantly associated with higher age (*P* = 0.001), myopic refractive error (*P* = 0.001), larger beta zone of parapapillary atrophy (*P*<0.001), presence of glaucomatous optic neuropathy (*P* = 0.007), presence of non-glaucomatous optic nerve damage (*P* = 0.001), and presence of diabetic retinopathy (*P* = 0.002). These significant associations remained to be statistically significant if, although it was already a multivariable analysis, a Bonferroni correction was performed to adjust for performing multiple statistical comparisons.

## Discussion

In our population-based study on subjects aged 50+ years, localized RNFLDs were detected in a frequency of 9.9±0.4% per eye and of 14.8±0.6% per subject. Localized RNFLDs were seen more often in the superior quadrant and inferior quadrant than in the temporal quadrant and nasal quadrant. Prevalence of localized RNFLDs increased with higher age, myopic refractive error, and larger parapapillary beta zone. Major ocular diseases associated with localized RNFLs were glaucoma, non-glaucomatous optic nerve damage and diabetic retinopathy.

The finding that localized RNFLDs were associated with disc hemorrhages in the univariate analysis is in agreement with clinical studies which showed a spatial relationship between localized RNFLDs and disc hemorrhages. The disc hemorrhages were usually located at the border of the RNFLD [25]. Since disc hemorrhages are significantly associated with glaucomatous optic neuropathy, the association between disc hemorrhages and RNFLDs was no longer significant after adjustment for the presence of glaucoma in the multivariate analysis in our study. The association between RNFLDs and myopic refractive error (or as a corollary, with axial length) is parallel to the association between a decreasing overall thickness of the RNFL and myopic refractive error [26]. The relationship between a higher prevalence of RNFLDs and older age may be due to the age-related increased prevalence of diseases, besides diabetic retinopathy, glaucoma, and non-glaucomatous optic nerve damage, which affect the RNFL and which were not assessed in our study, and/or it may also be due to the age-related loss of retinal cells and retinal ganglion cell axons [27]–[29]. Interestingly, RNFLDs were associated with a larger parapapillary beta zone, even after adjustment for the presence of glaucomatous optic neuropathy. This finding may have been caused by an optical artifact, since the background reflectivity is increased in parapapillary beta zone; or it may have been caused by large myopic peripapillary crescents in highly myopic eyes. A myopic crescent is conventionally measured as parapapillary beta zone. The association between RNFLDs and diabetic retinopathy can be explained by the occurrence of cotton wool spots in diabetic retinopathy. Cotton-wool spots as localized microinfarcts in the RNFL lead to localized RNFLDs [12].

The RNFLDs were defined by the contour line of the RNFL thickness on the OCT printouts. Although we assessed the presence of the localized RNFLDs manually, future studies may address the possibility of an automatic delineation of localized RNFLDs by the OCT device. It may be a step towards a (semi-)automatic assessment of the RNFL for the detection of optic nerve diseases and retinal diseases affecting the RNFL.

Potential limitations of our study should be mentioned. First, a major concern in any prevalence study is nonparticipation. The Beijing Eye Study 2011 had a reasonable response rate of 78.8%, however, differences between participants and non-participants could have led to a selection artifact. Second, the definition of a localized RNFLD included as maximal width a length of up to 180° on the printouts. Usually, localized RNFLDs as detected by ophthalmoscopy have a width of 5° to 45° [5]. The median width of the RNFLDs in our study was 24.6°, so that most of the RNFLDs assessed in our study represented the typical RNFLDs as seen upon ophthalmoscopy [30]. Third, the findings in our study can only be applied to subjects and patients over 50 years.

In conclusion, in our population-based study on Chinese subjects aged 50+ years, OCT-defined localized RNFLDs were present in 9.9±0.4% of the eyes or in 14.8±0.6% of the subjects. The prevalence of the RNFLDs increased with higher age, myopic refractive error, and larger parapapillary beta zone. Major ocular diseases associated with localized RNFLs were glaucoma, non-glaucomatous optic nerve damage and diabetic retinopathy. These data may be helpful for a semiautomatic assessment of the RNFL.
